# Not all cells are equal: effects of temperature and sex on the size of different cell types in the Madagascar ground gecko *Paroedura picta*

**DOI:** 10.1242/bio.025817

**Published:** 2017-06-19

**Authors:** Marcin Czarnoleski, Anna Maria Labecka, Zuzana Starostová, Anna Sikorska, Elżbieta Bonda-Ostaszewska, Katarzyna Woch, Lukáš Kubička, Lukáš Kratochvíl, Jan Kozlowski

**Affiliations:** 1 Jagiellonian University, Institute of Environmental Sciences, Gronostajowa 7, 30-387 Kraków, Poland; 2 Charles University, Department of Zoology, Viničná 7, 128 44 Praha, Czech Republic; 3 University of Białystok, Institute of Biology, Ciołkowskiego 1J, 15-245 Białystok, Poland; 4 Charles University, Department of Ecology, Viničná 7, 128 44 Praha, Czech Republic

**Keywords:** Life history, Optimal cell size, Phenotypic plasticity, Sexual dimorphism, Temperature-size rule

## Abstract

Cell size plays a role in evolutionary and phenotypically plastic changes in body size. To examine this role, we measured the sizes of seven cell types of geckos (*Paroedura picta*) reared at three constant temperatures (24, 27, and 30°C). Our results show that the cell size varies according to the body size, sex and developmental temperature, but the pattern of this variance depends on the cell type. We identified three groups of cell types, and the cell sizes changed in a coordinated manner within each group. Larger geckos had larger erythrocytes, striated muscle cells and hepatocytes (our first cell group), but their renal proximal tubule cells and duodenal enterocytes (our second cell group), as well as tracheal chondrocytes and epithelial skin cells (our third cell group), were largely unrelated to the body size. For six cell types, we also measured the nuclei and found that larger cells had larger nuclei. The relative sizes of the nuclei were not invariant but varied in a complex manner with temperature and sex. In conclusion, we provide evidence suggesting that changes in cell size might be commonly involved in the origin of thermal and sexual differences in adult size. A recent theory predicts that smaller cells speed up metabolism but demand more energy for their maintenance; consequently, the cell size matches the metabolic demand and supply, which in ectotherms, largely depends on the thermal conditions. The complex thermal dependency of cell size in geckos suggests that further advancements in understanding the adaptive value of cell size requires the consideration of tissue-specific demand/supply conditions.

## Abstract (precis)

**Summary:** The cell sizes of Madagascar ground geckos (*Paroedura picta*) vary according to body size, sex and developmental temperature, and the pattern of these differences depends on the cell type.

## INTRODUCTION

The body size achieved at maturation has fundamental effects on the evolutionary fitness of an organism (Ejsmond et al., 2015; Kozlowski, 1992, 2006; Stearns, 1992). In ectotherms, the size at maturation is highly sensitive to developmental temperatures, and it often decreases in warmer environments; this is called the temperature-size rule (Atkinson, 1994; Klok and Harrison, 2013). A recent reanalysis of published data on arthropods suggests that species with plastic responses that follow the temperature-size rule are more likely to have larger body sizes at higher latitudes, contributing to the geographic pattern called Bergmann's cline (Horne et al., 2015). Mechanistically, a change in body size requires a change in cell number and/or cell size, and there is evidence that body size and cell size undergo coordinated evolutionary (Arendt, 2007; Starostová et al., 2005; Stevenson et al., 1995) or phenotypically plastic changes (Arendt, 2007; Czarnoleski et al., 2013; Hessen et al., 2013). Evidence also shows that ontogenetic growth patterns might be associated with changes in cell number and cell size (Davison, 1956; Frýdlová et al., 2013; Starostová et al., 2013). As suggested by the theory of optimal cell size (TOCS), changes in cell number and cell size may not have equal fitness consequences, and the ultimate size of cells in organs results from a compromise between costs and benefits (Czarnoleski et al., 2015b; Davison, 1956; Kozlowski et al., 2003; Szarski, 1983). Following this theory, a body composed of many small cells is rich in cell membranes because with the decreasing volume of single cells, the total surface area of cells increases. Consequently, a tissue built of small cells expends more energy on membrane remodelling and the maintenance of ionic gradients across the cell surface. Moreover, the large overall cell membrane surface area increases the exchange of oxygen, nutrients and metabolites between the internal and external environments of the cells. Furthermore, the presence of many small cells in a tissue shortens the diffusion distances within cells and increases access to transcription sites due to the higher density of nuclei in the tissues (Czarnoleski et al., 2015a).

Considering the costs and benefits associated with cell size, ectotherms with increased demands for ATP in relation to the supply of oxygen, e.g., those in warm or hypoxic environments, are expected to consist of smaller cells (Atkinson et al., 2006; Czarnoleski et al., 2015a,b; Walczyńska et al., 2015). Addressing this hypothesis, we studied cell size and cessation of growth in Madagascar ground geckos (*Paroedura picta*, Peters 1854) reared from egg to adulthood in three different thermal environments. The animals originated from an experiment by Starostová et al. (2010), which demonstrated that male geckos grew larger than females at all temperatures and that geckos tended to attain the largest body size at the intermediate temperature. Furthermore, the geckos reared at the intermediate temperature produced the largest eggs (Starostová et al., 2012). Based on the TOCS, we predicted that geckos developed in warmer environments would have smaller cells than geckos developed in colder environments. Addressing the role of cell size changes in the cellular mechanisms related to body size changes, we also hypothesized that cell size changes were involved in the origin of the thermal- and sex-dependence of body size. To infer organism-wide trends in cell sizes, the majority of research on this topic has focused on singe cell types, e.g., erythrocytes in vertebrates (Gregory, 2001a,b; Starostová et al., 2005), or proxies of cell size, e.g., ommatidia in insects (Chown et al., 2007; Schramm et al., 2015), assuming that cell size is developmentally coordinated among different tissues in the body, as suggested by earlier studies (Heinrich et al., 2011; Kozlowski et al., 2010; Stevenson et al., 1995). However, there are counterexamples indicating the need to further examine the organism-wide coordination of cell size changes (Czarnoleski et al., 2016; Kozlowski et al., 2010). Therefore, in our study, we measured the sizes of different cell types together with the sizes of their nuclei. This way, we addressed whether cell size changes showed organism-wide coordination, and whether the relative sizes of cell nuclei stayed invariant. The invariance of the relative size of cells and their nuclei has long been a cytological puzzle, but it is believed to be rooted in the links between transcription, translation and nucleus size (Cavalier-Smith, 2005). Following Maciak et al. (2014), we expected invariance in the relative sizes of nuclei in cell types with high levels of anabolic activity but not in other cell types.

## RESULTS

Table S1 reports the means of raw values of body size, cell size and nuclei size in each group of geckos. Generally, the cell size within a given cell type varied considerably among individual geckos. Integrating this variance with data on body size, our factor analysis extracted three components, which together accounted for the 68% of the variance in the data (Table 1), showing that cell types clustered into three groups (components F1, F2 and F3). Overall, these results indicate that cell sizes in different cell types co-varied, and much of this variance was linked to differences in gecko body size. The variance in body size (snout-to-vent length, SVL) was mainly described by F1; geckos with higher F1 scores had longer SVLs and at the same time had larger erythrocytes, striated muscle cells and hepatocytes. Body size had a much smaller impact on F2 and F3; therefore, in making general conclusions, we consider F2 and F3 as being marginally associated with the variance in SVL. The most apparent trend is that cell sizes in the kidneys and duodenum were positively correlated with F2 (higher F2 scores indicate larger cells in these two tissues), whereas the sizes of epithelial skin cells and chondrocytes correlated positively with F3 (higher F3 scores indicate larger cells for these two types).

**Table 1.**
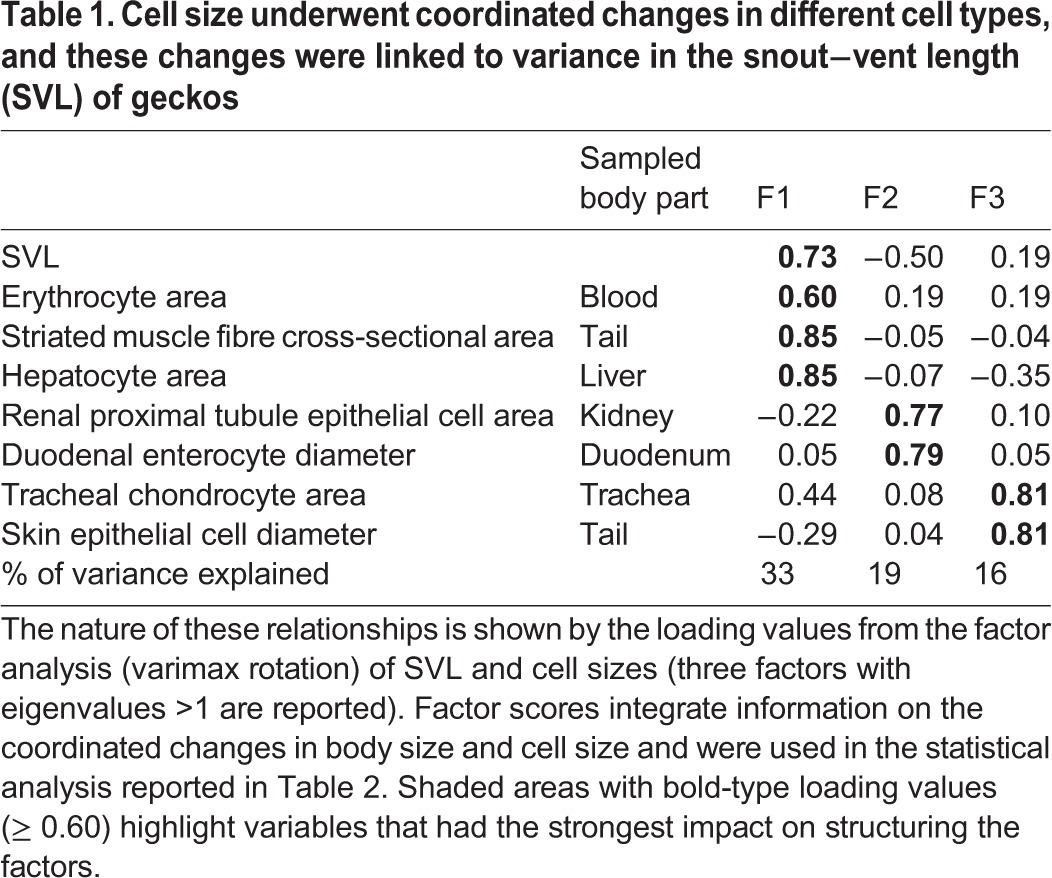
**Cell size underwent coordinated changes in different cell types, and these changes were linked to variance in the snout−vent length (SVL) of geckos**

**Fig. 1.**
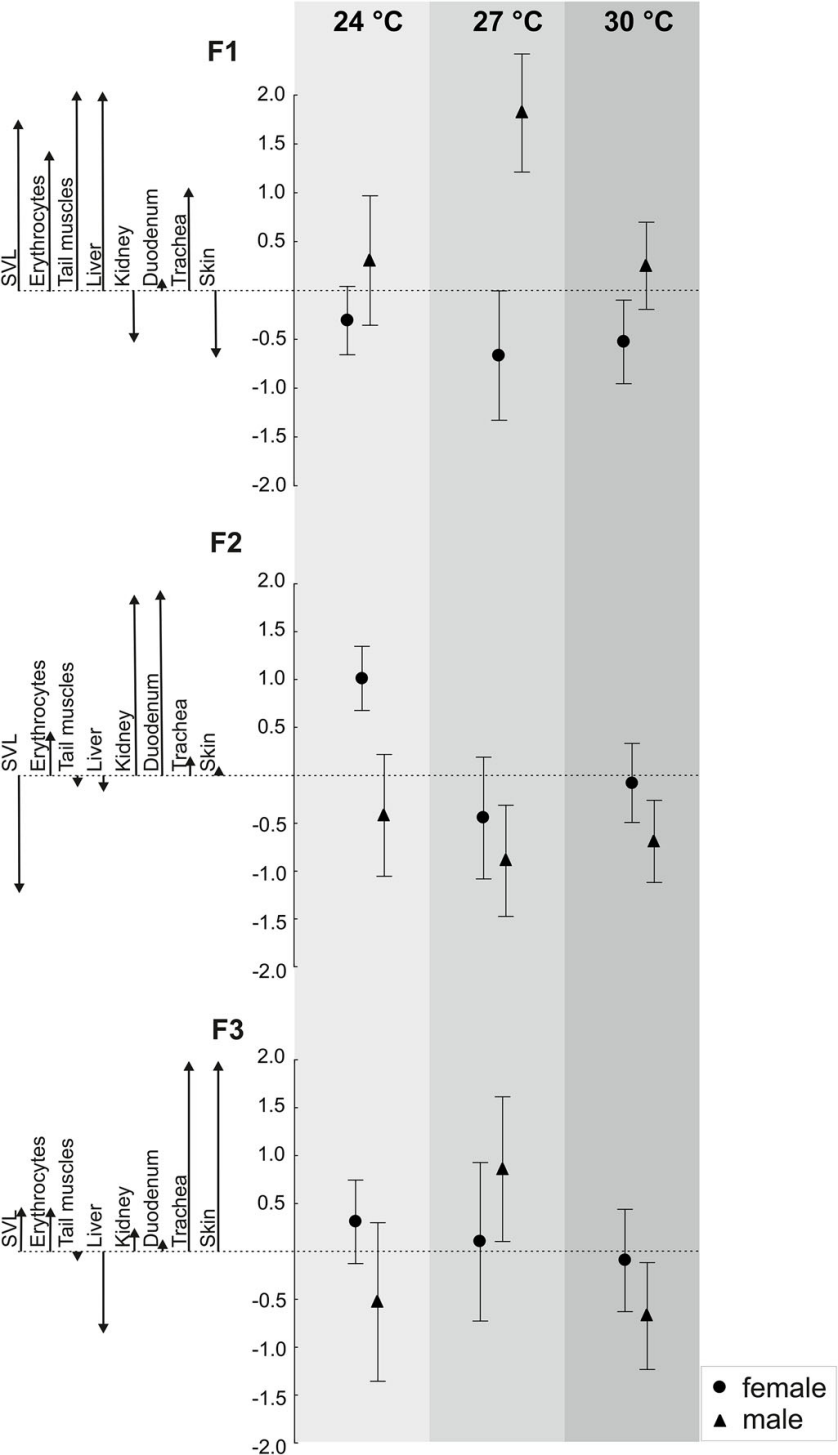
Rearing temperature and sex affected the cell size and snout vent length (SVL) of geckos, but the pattern of this dependence varied among cell types. Means with confidence intervals were estimated using GLM analyses performed on the scores of three factors produced by a factor analysis of cell size and SVL (see Table 2). Each factor, F1, F2 and F3, integrates information about SVL and the sizes of different cell types. The vertical axis indicates the factor score values. The arrows indicate the factor loading values reported in Table 1.

The general linear model (GLM) analysis of F1 scores (Table 2) showed a significant interaction between sex and temperature. Given that F1 was structured not only by cell size but also by body size, this result indicates that the sizes of the cell types involved in F1 played a significant role in the origin of the sexual and thermal dependence of body size. Overall, males had larger F1 scores than females, indicating larger body sizes (SVLs), erythrocytes, muscle cells and hepatocytes in males (Fig. 1). The significant sex × temperature interaction indicates that the magnitude of these sex differences depended on the thermal environment (Table 2). Generally, as shown in Fig. 1A, F1 scores were insensitive to temperature in females, but in males, they increased at the intermediate temperature (27°C), indicating that males developing at this temperature had especially longer SVLs and larger erythrocytes, muscle cells and hepatocytes.

**Table 2.**
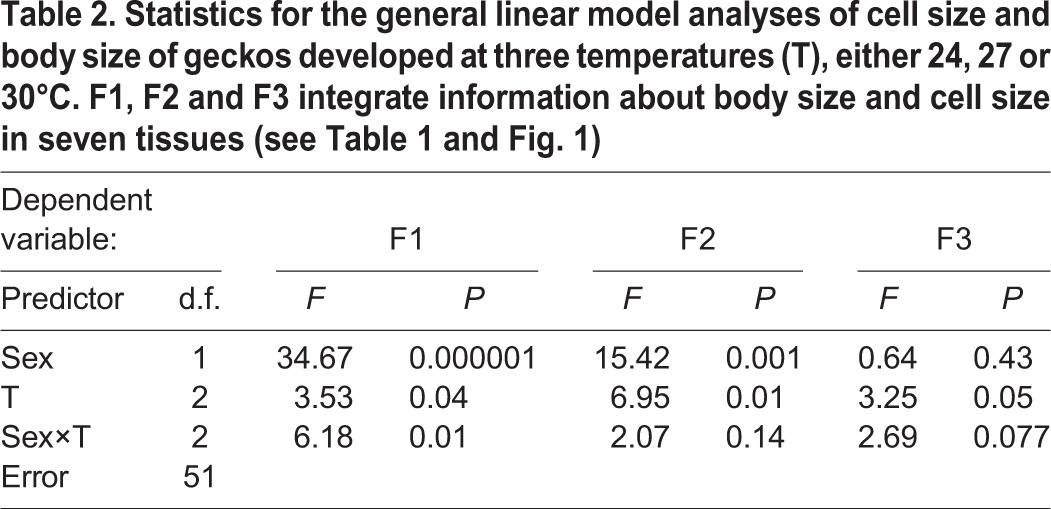
**Statistics for the general linear model analyses of cell size and body size of geckos developed at three temperatures (T), either 24, 27 or 30°C. F1, F2 and F3 integrate information about body size and cell size in seven tissues (see** **Table 1** **and** **Fig. 1)**

The GLM analysis of F2 scores (Table 2) shows that sex and the thermal developmental environment had significant impacts on F2 scores, but the sex × thermal environment interaction was not significant. Across all temperatures, males had smaller F2 scores than females (Fig. 1), indicating smaller renal proximal tubule cells and duodenal enterocytes in males. Both in males and females, F2 scores decreased at the intermediate temperature (27°C), indicating that geckos developing at this temperature had especially small renal proximal tubule cells and duodenal enterocytes (Fig. 1).

The GLM analysis of F3 scores (Table 2) shows that temperature had a significant effect on the size of skin epithelial cells and chondrocytes, but because the interaction between sex and the thermal environment was close to significance (*P*=0.077), we interpreted the effects of temperature in association with the sex of the geckos. As shown in Fig. 1, males had higher F3 scores and hence larger epithelial cells and chondrocytes at the intermediate temperature. This pattern resembled the pattern in F1 scores because the F3 scores were temperature-sensitive in males but relatively insensitive to temperature in females.

The GLM analysis of nucleus size (Table 3) shows that in all cell types, larger cells had larger nuclei. When compared at an average cell size, females had smaller nuclei in skin epithelial cells and duodenal enterocytes but larger nuclei in kidney cells. Females and males had similarly sized nuclei in erythrocytes, hepatocytes and chondrocytes. Warmer developmental conditions resulted in proportionally smaller nuclei in erythrocytes, kidney cells and duodenal enterocytes and larger nuclei in hepatocytes. The relative size of a nucleus did not change with temperature in chondrocytes and skin epithelial cells.

**Table 3.**
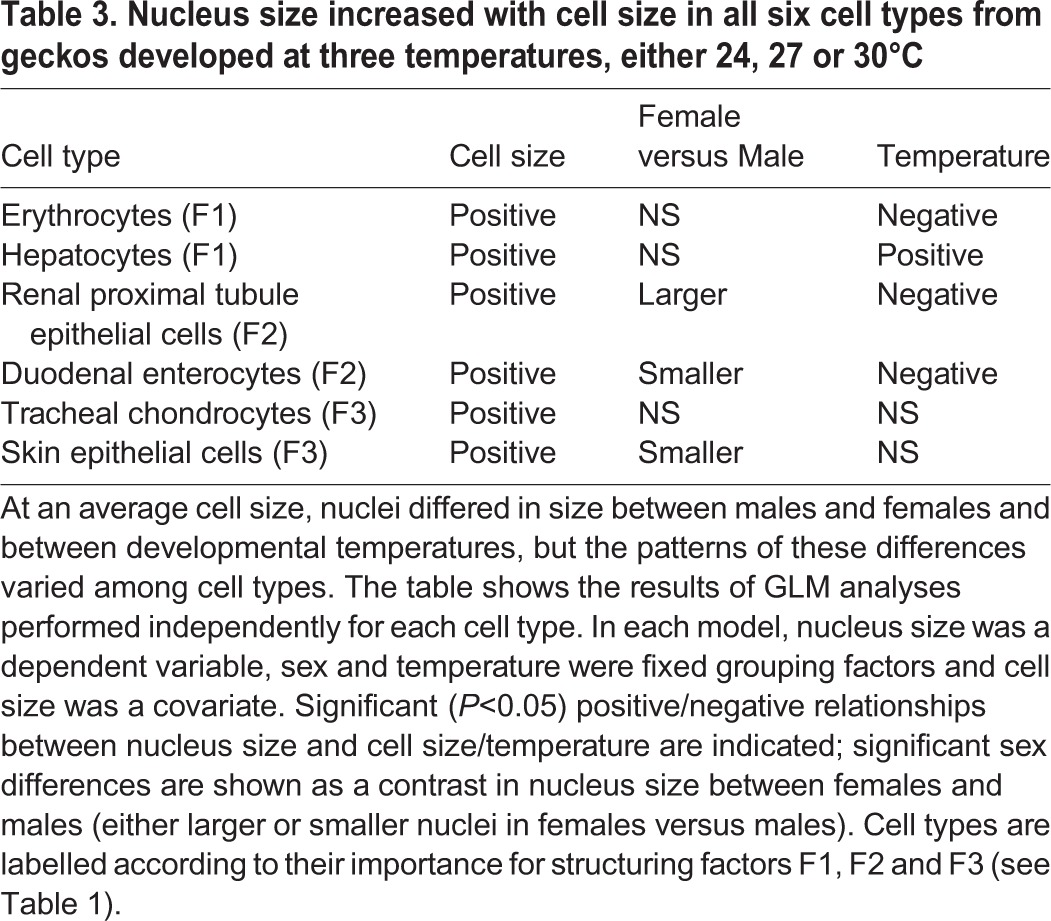
**Nucleus size increased with cell size in all six cell types from geckos developed at three temperatures, either 24, 27 or 30°C**

## DISCUSSION

Some theories assume that cell size stays invariant among organisms (West et al., 1997); however, we found that among adult *P. picta* geckos, the cell size varied according to body size, sex and thermal environment. Previous research on geckos suggests that interspecific differences in body size evolved partially through changes in cell size (Starostová et al., 2005). Our results indicate that cell size changes might also be involved in the evolution of sexual size dimorphism in geckos and in the plastic responses of geckos to the environment. Understanding the ecological and evolutionary implications of sexual size dimorphism is one of the central issues in evolutionary ecology (Dale et al., 2007; Horne et al., 2015; Reeve and Fairbairn, 2001; Stillwell et al., 2010; Teder and Tammaru, 2005), and our results suggest that the consideration of changes in cell size can benefit studies of sexual size dimorphism. In all of our thermal treatments, *P. picta* males consistently grew to a larger final body size than females, and this effect was associated with larger erythrocytes, muscle fibres and hepatocytes. Other cell types also differed between males and females, but we did not find evidence that these differences were directly related to sexual body size dimorphism. There is little published information on sex differences in the cellular architecture of the body in other organisms, particularly in the context of sexual body size dimorphism, although some work has been conducted with a fruit fly and a solitary bee (Adrian et al., 2016; Czarnoleski et al., 2013; Jalal et al., 2015; Kierat et al., 2017). Interestingly, the magnitudes of the sex differences in body and cell sizes in *P. picta* varied with rearing temperature, primarily due to the higher degree of phenotypic plasticity in males. In general, geckos maintained at the intermediate temperature (27°C) were characterised by the largest sex differences in body and cell sizes. This finding has important implications for future research, particularly considering that recent research on arthropods suggests that sexual differences in the thermal plasticity of adult size might not be widespread in nature (Horne et al., 2015). Based on the life history theory (Kozlowski, 2006; Kozlowski et al., 2004; Kozlowski, 1992; Stearns, 1992), we speculate that the thermal dependence of sexual size dimorphism in *P. picta* indicates different life history optima for males and females, which either diverge or converge depending on the thermal environment. Furthermore, the TOCS (Atkinson et al., 2006; Czarnoleski et al., 2013; Kozlowski et al., 2003; Szarski, 1983) suggests that sex differences in the thermal plasticity of cell size should correspond to sexual differences in thermal performance.

A coupling between cell size and body size has been reported previously in a range of different organisms, including rotifers (Czarnoleski et al., 2015b; Walczyńska et al., 2015), flies (Arendt, 2007; Czarnoleski et al., 2013; Stevenson et al., 1995), crustaceans (Davison, 1956; Hessen et al., 2013), snails (Czarnoleski et al., 2016), lizards (Starostová et al., 2005), birds and mammals (Kozlowski et al., 2010). Altogether, our results and this previous evidence suggest that cell size is not invariant and that ignoring this variation might prevent a full understanding of the origin of body size diversity at the inter- and intra-specific levels. Van Voorhies (1996) envisioned that variance in cell size and its environmental dependence drives the origin of enigmatic patterns in the thermal dependence of ectotherm adult size, such as the temperature-size rule (Atkinson, 1994) and Bergmann's clines (Ashton and Feldman, 2003). The adult body size of the geckos studied here changed plastically with rearing temperature, although not in a systematic manner (for details see Starostová et al., 2010), and our data show that these changes were tightly coupled with changes in cell size in some tissues. Nevertheless, given the fitness consequences related to adult size (Kozlowski, 2006; Kozlowski, 1992; Stearns, 1992), it is hard to imagine that natural selection would not overcome developmental limitations that entirely link the fate of body size to changes in cell size as implicated by the ideas of Van Voorhies (1996). Our data on geckos show that individual differences in adult body size cannot be entirely explained by the variance in cell size, which clearly indicates that a difference in body size is not an unavoidable mechanistic consequence of changes in cell size (and vice versa). In support of this, Adrian et al. (2016) demonstrated that fruit flies subjected to experimental evolution diverged genetically in cell size and body size between different thermal environments, and most of the genetic variation in cell size was independent of the variation in body size. Certainly, independent changes in body size and cell size are greatly restricted in eutelic organisms, which have a fixed number of cells, such as rotifers, tardigrades or nematodes (Czarnoleski et al., 2015b; Walczyńska et al., 2015). Nevertheless, emerging evidence suggests that strict eutely might be less common than previously thought (Cunha et al., 1999), and even in a strictly eutelic organism, cells can fuse to form larger syncytia, which alleviates the mechanistic dependence between cell size and body size (Czarnoleski et al., 2015b).

To date, the majority of research on the variance of cell size has focused on single cell types, assuming that other cells in a body change size in unison (Arendt, 2007; Czarnoleski et al., 2015a; Frýdlová et al., 2013; Gregory, 2001b; Maciak et al., 2011; Starostová et al., 2005). At this stage, we do not know of a precise molecular mechanism that might determine the organism-wide coordination of cell sizes, although recent advancements in cell biology suggest the role of TOR/insulin signalling pathways (De Virgilio and Loewith, 2006; Grewal, 2009). In agreement with the idea of coordinated changes in cell size, we found that if a gecko had larger cells in one tissue, it tended to also have larger cells in some other tissues. Studies of other species provide similar evidence wherever sufficient data has been collected (Azevedo et al., 2002; Czarnoleski et al., 2016; Heinrich et al., 2011). Additionally, comparative evidence in plants (Brodribb et al., 2013), flies (Stevenson et al., 1995), amphibians and birds (Kozlowski et al., 2010) suggests that evolutionary changes in the size of different cell types proceed in a coordinated manner. Nevertheless, our data on geckos pose some uncertainty because not all seven cell types studied changed their size in a completely coordinated manner; instead, they clustered in three groups according to the pattern of the sex and thermal dependence of cell size. It is of interest that the size of erythrocytes matched the size of hepatocytes and the fibres of striated muscles but not the size of the other cell types. This finding brings into question the assumption of many studies (e.g. Gregory, 2001a; Starostová et al., 2013) that differences in the sizes of erythrocytes reflect differences in the sizes of other cell types. The generality of such findings needs to be determined before re-evaluating the hypothesis of coordinated changes in cell size; however, existing evidence suggests that the phenomenon might not be rare. Evidence suggesting such clustering has been found in a bryozoan species (Atkinson et al., 2006), two subspecies of a snail (Czarnoleski et al., 2016), and laboratory mice (Maciak et al., 2014) and interspecifically in mammals (Kozlowski et al., 2010), rodents and gallinaceous birds (D. Dragosz-Kluska, T. Pis, K. Pawlik, F. Kapustka, W. Kilarski, M.C., A.M.L., J.K., unpublished data); however, such evidence is lacking in flies (Azevedo et al., 2002; Heinrich et al., 2011; Stevenson et al., 1995), plants (Brodribb et al., 2013), birds and amphibians (Kozlowski et al., 2010). Why and how cell size changes might be coordinated in a tissue-specific manner remain open questions. We expect that this phenomenon might reflect the matching of differences in physiological activity among tissues (Maciak et al., 2014) and differences among tissues in the tendency to undergo somatic polyploidy. Some evidence suggests that changes in somatic ploidy can contribute to the origin of the thermal responses of cell size (Hessen et al., 2013; Jalal et al., 2013, 2015).

Despite the apparent connections among developmental environment, cell size and metabolic rate (Czarnoleski et al., 2013, 2015b; Davison, 1956; Heinrich et al., 2011; Hermaniuk et al., 2017; Hessen et al., 2013; Maciak et al., 2011; Starostová et al., 2013), we remain far from a full understanding of how cell size affects organismal performance in nature. We found that the size of cells in geckos is sensitive to the thermal conditions during development, but the pattern of this dependence is only in partial agreement with the predictions of the TOCS. Consistent with the idea that smaller cells help meet the increased metabolic demand of a warm ectotherm, geckos developing in warmer conditions have smaller cells of all seven cell types. However, in disagreement with the predictions, the thermal-dependence of cell size changes in a complex tissue-specific manner according to the range of temperatures and the sex of the animals. If, as predicted by the TOCS, cell size in a tissue is optimized according to metabolic demand and the supply of oxygen and resources, the complexity found in this study suggests that the balance between demand and supply changes among organs in a sex-dependent manner. In fact, organs differ dramatically in their physiological workload and blood supply, and sexes are likely to have different physiological profiles. For example, the liver performs exceptionally intense anabolic and catabolic work, and all hepatocytes are in a direct contact with hepatic capillaries that are supplied with blood via a dual perfusion system (Kerr, 2010). Our view is indirectly supported by the evidence from geckos' cell nuclei. In conflict with the cytological postulate regarding the invariance in karyoplasmic ratios (Cavalier-Smith, 2005), we found that the relative size of cell nuclei changed with developmental temperature and sex and that the nature of these changes was tissue specific. Maciak et al. (2014) suggested that molecular crowding in the nucleus and cytoplasm affects transcription and translation rates; therefore, a change in the relative volume of the nucleus and cytoplasm should indicate changes in translational and transcriptional activity. In the studied geckos, the relative size of a nucleus was independent of temperature in cells more involved in body structures, such as chondrocytes and skin epithelial cells, suggesting that translational activity in these cells was insensitive to temperature. In contrast, in cell types more involved in supracellular functions, the relative size of the nuclei either decreased (erythrocytes, renal cells and duodenal cells) or increased (hepatocytes) with temperature, suggesting negative and positive effects, respectively, of temperature on translation rates.

It is usually difficult to extrapolate discoveries from simple experiments, such as ours, to the complexity of organisms living in natural ecosystems. Nevertheless, our evidence points to changes in cell size as part of the life history strategy of organisms; therefore, considering the role of cell size can help us better understand different ecological and evolutionary phenomena, such as the origin of the thermal plasticity of body size and body size sexual dimorphism. The TOCS helps to view the complex nature of cell size patterns in *P. picta* as a manifestation of a single phenomenon – matching the size of cells to the metabolic demand of the tissue and its supply of oxygen and resources. An important message from our study is that advancements in research on the fitness consequences of cell size are in critical need of a deeper understanding of how demand and supply change between different tissues and how the biochemical activity of a cell depends on the relative volumes of its cytoplasm and nucleus.

## MATERIALS AND METHODS

### Animals and experimental design

The gecko species studied (*P. picta*) is a medium-sized nocturnal lizard endemic to Madagascar (Schönecker, 2008). Due to its rapid growth and development (Kubička et al., 2016; Starostová et al., 2010) and the availability of genetic information (Starostová and Musilová, 2016), it constitutes a convenient model organism for reptile studies. For the present study, we used fully grown adults of *P. picta* that were reared in the experiment conducted by Starostová et al. (2010). Briefly, Starostová et al. (2010) obtained eggs from 20 *P. picta* females maintained under common conditions and incubated them in thermal cabinets with a 12 h light:12 h dark cycle at one of three temperatures: 24, 27 or 30°C (±0.3°C). After hatching, the geckos were fed *ad libitum* twice a week, and the feed consisted of crickets powdered with vitamins and minerals. The growth of each animal was monitored until the clear cessation of growth. The snout-to-vent length (SVL) of each animal was then measured, and the animals were decapitated following anaesthesia (for more details, see Starostová et al., 2010). We then collected tissue samples for the present cell size analysis. Cell size can change through ontogeny in geckos (Starostová et al., 2013). By studying geckos that had reached their final body size, we ensured that all had matured and reached a similar life stage at which further cell growth was not expected.

### Histology and cell size

Altogether, we processed 57 geckos: 18 females and 5 males raised at 24°C, 5 females and 6 males raised at 27°C and 12 females and 11 males raised at 30°C. From each gecko, we took a blood sample and made a blood smear. The smears were used to measure erythrocytes. We also dissected samples of the trachea, kidneys, liver, duodenum and tail (a tip) from each animal, which were used to measure the sizes of the other six cell types. After drying, blood smears were fixed for 5 min in methanol (Penta, Czech Republic) and stained with Gill II haematoxylin (Carl Roth, Germany) and a 1% ethanol solution of eosin Y (Analab, Poland). The samples of other tissues were fixed for 24 h in 10% buffered neutral formalin (Penta, Czech Republic) and then transferred to 70% ethanol (Lach-Ner, Czech Republic). After dehydration in graded series of ethanol (Linegal, Poland), cleared in ST Ultra (Leica, Germany), the tissue samples were embedded in Paraplast Plus (Leica). Serial cross sections (4-µm thick) were cut with a rotary microtome Hyrax M55 (Zeiss, Germany). Slides with tracheal samples were stained with Alcian Blue (Carl Roth) and Nuclear Red (Carl Roth). Slides with other tissue sample types were stained with Ehrlich haematoxylin (Carl Roth) and a 1% eosin Y solution in ethanol. Blood smears were photographed under a light microscope (Eclipse 80i, Nikon, Japan) equipped with an Axio Cam MRc5 (Zeiss) digital camera and ZEN (Zeiss) software. Slides with other tissue types were digitalized under an automatic light microscope (BX51, Olympus, Japan) equipped with an XC10 (Olympus) digital camera and dotSlide (Olympus) software or a VC50 (Olympus) digital camera and VS-120 (Olympus) software. Erythrocytes were measured using ImageJ software (National Institute of Health, USA) and other cell types were measured using CellSens (Olympus).

We measured the sizes of seven cell types, and in six cell types we also measured their nuclei. Assuming nuclei were elliptical in shape, the area of each nucleus was calculated based on its long and short axes. The sizes of erythrocytes, chondrocytes and striated muscle fibres were determined for individual cells. The areas of erythrocytes were calculated from their two perpendicular diameters, assuming an elliptic shape of these cells. The areas of chondrocytes were calculated based on the outlines of lacunae. When chondrocytes formed isogenic groups, we measured one chondrocyte per group. To measure striated muscle fibres, we outlined cross-sections of individual fibres and calculated the areas of the cross-sections. Note that a fibre of striated muscles is a syncytium formed by the fusion of multiple cells. Cell membranes were not clearly visible in liver, kidney proximal tubule, duodenum and skin epithelium samples, which prevented reliable measurements of individual cells. In these tissues, cell size was estimated based on the measured areas of cell groups. Cross sections of liver were divided into sectors, and around the central point of each sector, we outlined a circular area of approximately 4000 µm^2^ (the actual size of the area was then used in cell size calculations). The mean size of hepatocytes (µm^2^) in each gecko was calculated by dividing the total areas of all circles by the total number of nuclei observed within the areas. Nuclei located on area borders were included if the majority of their surface was inside an outlined area. Note that if a nucleus could not be clearly classified as being located inside or outside the area, it was given a 50% chance of being classified as being located inside the area. For kidney samples, we measured the areas of proximal tubule cross-sections. When we were unable to measure the whole cross-section, we focused on a well-defined area with at least two visible nuclei. We counted the nuclei within each area, and after summing all of the areas and the number of nuclei for each individual, we calculated the mean area of renal proximal tubule epithelial cells per individual (µm^2^). To assess the size of epithelial cells in duodenum mucous membranes and in the basal layer of the skin, we identified groups of adjacent nuclei that were aligned linearly in transects. Following the methods of Wieczorek et al. (2015) and Czarnoleski et al. (2016), we measured the distance between nuclei at the two ends of each transect (µm). The mean cell size for an individual gecko was calculated by dividing the total length of the transects by the total number of nuclei associated with these transects.

In total, we obtained the following number of measurements per individual gecko: 59‒71 erythrocytes in blood smears (with nuclei); 99‒101 striated muscle fibres in tail samples (no nuclei measured); 100 cell groups of duodenal enterocytes, with 100 nuclei; 36‒72 groups of skin epithelial cells in tail samples, with 15‒69 nuclei; 100 renal proximal tubules, with 100 nuclei; 60 tracheal chondrocytes, with 60 nuclei; and 50 groups of hepatocytes, with 50 nuclei.

### Statistical analysis

All statistical analyses of cell size were performed on mean values calculated for each gecko and cell type. To integrate information on different cell types and body sizes, we performed a factor analysis on cell sizes for different cell types and body size (SVL). We first extracted principal components with eigenvalues >1 and then rotated them using a varimax procedure. Scores for these factors were used as a measure of coordinated changes in body size and cell size. The scores were analysed using a general linear model (GLM; Statistica 12, StatSoft, Poland) that included sex, developmental temperature and an interaction between sex and temperature as fixed factors. The results of this model were used to test the hypotheses about the dependence of cell size on thermal environment and sex. The invariance of the relative sizes of nuclei was tested using a GLM (separate model for each cell type) that included sex and temperature as fixed factors and cell size as a numeric covariate.

### Ethics

The study was conducted with the approval of the Ethical Committee of the Faculty of Science, Charles University, Prague, and The Ministry of Education, Youth and Sports of the Czech Republic (Permit Number 34709/2010-30).
